# Evaluation of a Serious Self-Regulation Game Intervention for Overweight-Related Behaviors (“Balance It”): A Pilot Study

**DOI:** 10.2196/jmir.4964

**Published:** 2016-09-26

**Authors:** Jorinde Spook, Theo Paulussen, Gerjo Kok, Pepijn van Empelen

**Affiliations:** ^1^ TNO Life Style Leiden Netherlands; ^2^ Maastricht University Department of Work and Social Psychology Maastricht University Maastricht Netherlands; ^3^ Wageningen University and Research Department of Communication, Philosophy and Technology: Centre for Integrative Development Wageningen Netherlands

**Keywords:** Balance It, effect evaluation, serious game, self-regulation, prevention and control, health promotion, dietary intake, physical activity

## Abstract

**Background:**

Serious games have the potential to promote health behavior. Because overweight is still a major issue among secondary vocational education students in the Netherlands, this study piloted the effects of “Balance It,” a serious self-regulation game intervention targeting students’ overweight-related behaviors: dietary intake and physical activity (PA).

**Objective:**

We aimed to pilot the effects of Balance It on secondary vocational education students’ dietary intake and PA.

**Methods:**

In total, 501 secondary vocational education students participated at baseline (intervention: n=250; control: n=251) in this pre-post cluster randomized trial. After 4 weeks, at immediate posttest, 231 students filled in the posttest questionnaire (intervention: n=105; control: n=126). The sample had a mean age of 17.28 (SD 1.26, range 15-21) years, 62.8% (145/231) were female, and 26.8% (62/231) had a non-Dutch background. Body mass index (BMI kg/m^2^) ranged from 14.4 to 31.1 (mean 21.1, SD 3.3). The intervention and control groups were compared on the primary (behavioral) outcomes of dietary intake (fruit and vegetable consumption, snack consumption, and soft drink consumption) and PA (moderate and vigorous). Additionally, we explored (1) differences between the intervention and control groups in determinants of dietary intake and PA, including attitude, self-efficacy, intention, barrier identification, action planning, and action control, and (2) differences between active (intervention) users and the control group in dietary intake, PA, and associated determinants.

**Results:**

After corrections for multiple testing, we did not find significant differences between the intervention group and control group in terms of dietary intake, PA, and determinants of dietary intake and PA. Exploratory research indicated that only 27.6% (29/105) of the intervention group reported actual intervention use (ie, active users). For exploratory reasons, we compared the active users (n=29) with the control group (n=124) and corrected for multiple testing. Results showed that active users’ snack consumption decreased more strongly (active users: mean change=–0.20; control group: mean change=–0.08; beta=–0.36, *P*=.01, *R*^2^ change=.05), and their use of active transport had a stronger increase (active users: mean change=0.92; control group=–0.12; beta=1.58, *P*=.02, *R*^2^ change=.03) than the control group. Results also revealed significant differences in action planning (active users: mean change=0.42; control group: mean change=0.07; beta=0.91, *P*=.01, *R*^2^ change=.04) and action control (active users: mean change=0.63; control group: mean change=–0.05; beta=1.25, *P*=.001, *R*^2^ change=.08) in terms of unhealthy eating.

**Conclusions:**

The Balance It intervention did not show favorable effects on dietary intake and PA compared to the control condition. However, only a small number of people in the intervention condition actually used Balance It (27.6%). Exploratory analyses did suggest that, if used as planned, Balance It could contribute to changing dietary intake and PA behaviors, albeit it remains debatable whether this would be sufficient to prevent overweight.

## Introduction

Overweight and obesity are related to various chronic health problems, including type 2 diabetes mellitus, cardiovascular disease, cancer, and also psychosocial problems [1-6]. In the Netherlands, approximately 20% of youth (aged 16-20 years) from low socioeconomic status (SES) families are overweight or obese [7], and prevalence is even higher among youth with Turkish or Moroccan descent [8-10]. Treatment of overweight remains a challenge; hence, it is important to target overweight-related behaviors (eg, dietary intake and physical activity [PA]) in intervention studies designed to prevent overweight in low SES youth.

Recent advances in technology enable researchers to tailor dietary intake and PA interventions to the needs of the target population. Moreover, it is possible to design a program that is cost effective, that has a wide reach, and that can function as a standalone program [11,12]. Reviews highlight the potential of computer-tailored interventions in terms of effectively changing and promoting health-related behaviors [13-15], yet targeting young people; immigrant groups; people with a low, primary, or basic vocational education; and people with weak health motivation can still be challenging [16]. To overcome hurdles such as low reach and limited adoption of computer-tailored interventions, several strategies have been recommended, including increasing the interactivity and visual attractiveness of the program [17-19]. Serious gaming is a promising method that can be used to stimulate intervention use because such games are designed to be highly enjoyable, attention grabbing, and intrinsically motivating [20-23]. In previous research [24], serious gaming interventions (eg, “Diab” and “Nano”) appeared to increase fruit and vegetable intake. However, playing these games did not increase water consumption, PA, or body composition. Thompson et al [25] indicated that action intentions may be an important component of successful interventions to stimulate youth fruit and vegetable intake [25], which in combination with coping plans may also account for PA [26]. As such, these studies showed that serious games have great potential to change health-related behaviors. However, according to DeSmet et al [19], serious games generally fall short in applying effective behavior change methods to change health-related behaviors. DeSmet et al [19] advocate the use of dual theoretical frameworks, stressing the importance of a theoretical foundation in both behavioral prediction and game theories. To this end, we combined effective behavior change techniques (as applied in computer-tailored interventions) with serious gaming strategies to encourage intervention use and target health behavior change simultaneously. As such, we developed a serious self-regulation game intervention called “Balance It.”

Balance It combines behavior change techniques derived from self-regulation theory [27,28] with serious game elements. It is a serious self-regulation game designed to target dietary intake and PA among secondary vocational education students. Balance It was systematically developed by means of Intervention Mapping, a protocol that enables the systematic planning of theory- and evidence-based interventions [29]. Further elaboration on the design rationale of Balance It can be found elsewhere [30]. Our key research objectives in this pilot study were to (1) identify the effectiveness of Balance It on changes in (determinants of) secondary vocational education students’ dietary intake and PA, and (2) evaluate the uptake and usage of the game and the game elements.

## Methods

### Study Design

A cluster randomized trial was conducted in 2014/2015 with measurements taken at baseline, immediately posttest (after 4 weeks of game play), and at a 4-week follow-up. Fifteen vocational education schools in the Netherlands were approached to participate in this study. In total, 4 schools agreed to participate and were randomly assigned to the intervention or waiting list control group. To counteract contamination effects between participant groups and to increase participants’ compliance with the study, random allocation to conditions took place at the level of schools [31]. All procedures were approved by The Research Ethics Board of the School of Psychology and Neuroscience (Maastricht University).

### Participants

The power calculation (alpha=.05, beta=.80) was based on a mean effect size of 31 for dietary intake and PA intervention [32]. This required a minimum of 130 participants for both the intervention and the control group. Students who did not have a mobile phone operating on iOS or Android were exempted from participation, as were students younger than 16 years or older than 21 years. Students younger than 16 years were exempted from participation because, within the Netherlands, individuals are allowed to provide informed consent from the age of 16 years. Participants older than 21 years were also exempted from participation because they had outgrown puberty and were not targeted by the intervention. All other students between the ages of 16 and 21 years were eligible for participation.

### Procedures

One week before the study, participants received passive consent forms addressed to their parents or caregivers. At baseline, a research assistant went to the schools to introduce the study and to collect the survey data. In total, 238 students gave their consent to participate in this study. At baseline, participants filled out an online baseline questionnaire regarding their mean dietary intake and PA, social cognitive factors (ie, attitude, self-efficacy, and intention), perceived barriers, self-regulation skills, action planning, and action control. After they finished the questionnaire, participants received a link to the Balance It website and further instructions about downloading the Balance It app from the research assistant. All students received a posttest questionnaire 4 weeks after the baseline measure was taken. Participatory incentives of €20 vouchers were randomly distributed among participants. The chance of winning a voucher increased with the number of measures completed (one measure 1:8, two measures 2:8).

### Balance It

**Figure 1:**
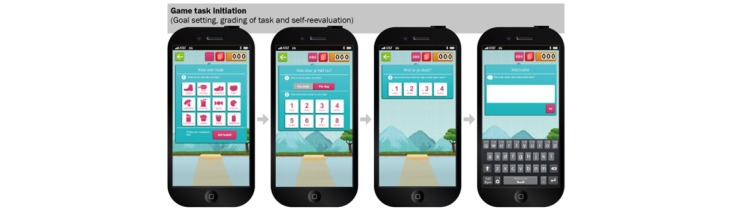
Screenshots of task initiation in the Balance It app.

Balance It was designed as a tailored, interactive multimedia game in which each game could be played either individually or competitively with others, at any time and place desired. It was designed as an educational, strategic game that could be played on a daily basis for 4 continuing weeks or on a weekly basis for 6 continuing weeks. Within each game, players set their own graded tasks (eg, to eat two pieces of fruit per day), which were selected from a multiple-choice list (Figure 1; [30]). They monitored and evaluated these goals on a daily or weekly basis, depending on the type of game they chose to play. Each day or week, players were prompted with their goals and reminded to return to the game. Visual feedback on self-reported goal attainment was provided for each goal, and players were prompted to reflect on their condition and on the perceived barriers or facilitators of goal accomplishment. Finally, participants were encouraged to formulate implementation intentions. In turn, these implementation intentions, or strategies, could be set as reminder prompts at any specific time point the player preferred. Information about formulating implementation intentions was provided on the Balance It website. The website also provided a general overview of the participant’s progress and a peer-support system (ie, the Balance It forum). Reinforcement was given in the form of obtainable “Tetris-shaped” building blocks and the allocation of “super powers” after goal accomplishment and self-evaluation of the targeted behavior. With these building blocks, players were encouraged to build a tower and to keep the tower in balance (see Figure 1; for a full description of the game design and content see [30]).

### Waiting List Control Group

At baseline, the control group was instructed to fill in the baseline questionnaire and informed that the researcher would return in 4 weeks for a posttest measure. Between measures, no interventions were offered by the researchers. Immediately following the posttest, students were provided with information about Balance It and given the opportunity to play.

### Behavioral Outcome Measures

#### Dietary Intake

The assessment of dietary intake was derived from a validated food frequency questionnaire [33]. Questions were related to the participant’s mean daily fruit and vegetable intake, snack consumption, and soft drink consumption. Answers were given on an 8-point scale on which participants could record the number of days they consumed specific foods, ranging from 0 (never or almost never) to 7 (every day). In addition, the quantity of their dietary intake was assessed. Response categories ranged from 1 (half portion or piece a day) to 7 (three or more portions or pieces a day). Based on these scores, the mean intake per day was calculated.

#### Physical Activity

The PA measures were derived from the Injuries and Physical Activity in the Netherlands (“Ongevallen en Bewegen in Nederland”) questionnaire (validated; [34]). Questions were related to the participant’s mean moderate PA (walking and cycling) and vigorous PA (exercise). For example, moderate PA was operationalized as “During the last week, how many days did you carry out 30 minutes of moderate PA?” Answers were given on an 8-point scale on which participants could rate the number of days they were moderately or vigorously active, ranging from 0=never or almost never to 7=every day.

### Determinants of Dietary Intake

In addition to the behavioral outcomes, social cognitive factors were measured for healthy dietary intake (fruit and vegetable intake) and unhealthy dietary intake (snacks, sweets, and soft drink consumption). All measures of determinants were preceded by a stem, followed by the behavioral outcome measures as subcategories.

#### Attitude

Attitudes toward dietary intake were assessed by three items using semantic differential response scales, such as “I think that eating two pieces of fruit a day is...” (1=very bad to 5=very good; 1=very unpleasant to 5=very pleasant; 1=very unhealthy to 5=very healthy) derived from [35,36]. Cronbach alpha for the healthy dietary intake attitude items was .87 and Cronbach alpha for the unhealthy dietary intake attitude items was .87.

#### Self-Efficacy

Self-efficacy toward dietary intake was assessed by one item preceded by a question stem: “If I want to, I am capable of...” Items were derived from van der Horst et al [35] and from Van Genugten et al [36]. Response options ranged from 1=definitely not to 5=definitely. Cronbach alpha for the healthy dietary intake self-efficacy items was .82. Cronbach alpha for the unhealthy dietary intake self-efficacy items was .88.

#### Intention

Dietary intake intention was assessed with one item preceded by the stem: “I planned to...” derived from [35,36]. Response options ranged from 1=definitely not to 5=definitely. Cronbach alpha for the healthy dietary intake intention items was .82. Cronbach alpha for the unhealthy dietary intake intention items was .93.

#### Barrier Identification

Barriers to healthy dietary intake were assessed separately from barriers to unhealthy dietary intake because different barriers influence fruit and vegetable intake and unhealthy dietary intake. Healthy dietary intake was assessed with five items using 5-point Likert scales: “I am capable of eating sufficient fruit and vegetables, also when I am...” Response options ranged from 1=definitely not to 5=definitely. Subcategories referred to when I am alone, during the weekend, when I am in a hurry, when I experience difficulties preparing fruits and vegetables, and when there is a lack of choice. Items were derived from previous measures [37,38]. Cronbach alpha for the healthy dietary intake barrier identification items was .86.

Barriers to unhealthy dietary intake were assessed with 13 items using 5-point Likert scales: “I am capable of eating a limited amount of unhealthy snacks, also when I am...” Response options ranged from 1=definitely not to 5=definitely. Subcategories referred to physical settings (eg, when I am at home), sedentary activities (eg, when I am watching TV), social settings (eg, when I am at a party), and mood (eg, when I am sad). Items were derived from previous measures [37,38]. Cronbach alpha of the unhealthy dietary intake barrier identification items was .96.

#### Action Planning

Action planning in terms of dietary intake was assessed by four items, such as “I have a clear plan for when I...” Response options ranged from 1=definitely not to 5=definitely. Subcategories referred to when, where, how, and how often participants planned to eat more healthy or less unhealthy foods (derived from [35,36]). Cronbach alpha for the healthy dietary intake action planning items was .97. Cronbach alpha for the unhealthy dietary intake action planning items was .96.

#### Action Control

Action control in terms of dietary intake was measured with four items using 5-point Likert scales, such as “During the last month, I have constantly monitored my...” Response options ranged from 1=definitely not to 5=definitely. Subcategories referred to self-monitoring of fruit and vegetable consumption, awareness of fruit and vegetable standards, self-regulatory effort to eat more healthy and less unhealthy foods, and self-regulatory effort to conform to norm behavior (eg, eat two pieces of fruits a day) (derived from [39]). Cronbach alpha for the healthy dietary intake action control items was .94. Cronbach alpha for the unhealthy dietary intake action control items was .94.

### Determinants of Physical Activity

Social cognitive factors were also measured for moderate PA (eg, walking and cycling) and vigorous PA (eg, exercising). All measures of PA determinants were preceded by a stem followed by the behavioral outcome measures subcategories.

#### Attitude

Attitudes toward PA was assessed by three items using semantic differential response scales, such as “I think that exercising is...” (1=very bad to 5=very good; 1=very unpleasant to 5=very pleasant; 1=very unhealthy to 5=very healthy) (derived from [35,36]). Cronbach alpha for the PA attitude items was .79.

#### Self-Efficacy

Self-efficacy toward PA was assessed by one item preceded by a question stem: “If I want to, I am capable of...” Items were derived from Van der Horst et al [35] and Van Genugten et al [36]. Response options ranged from 1=definitely not to 5=definitely. Cronbach alpha for the PA self-efficacy item was .75.

#### Intention

Intention was assessed with one item preceded by the stem: “I planned to...” (derived from [35,36]). Response options ranged from 1=definitely not to 5=definitely. Cronbach alpha for the PA intention item was .73.

#### Barrier Identification

Barriers to PA were assessed with seven items using 5-point Likert scales, such as “I am capable of being more physically active, also when I am...” Response options ranged from 1=definitely not to 5=definitely. Subcategories referred to when I am busy, when I am stressed, if I failed last time, when I am tired, when it is raining, if I do not have the time, and if I do not get social support (derived from [37,38]). Cronbach alpha for the PA barrier identification items was .93.

#### Action Planning

Action planning in terms of PA was assessed by four items using 5-point Likert scales, such as “I have a clear plan for when I...” Response options ranged from 1=definitely not to 5=definitely. Subcategories referred to when, where, how, and how often participants planned to be more physically active (derived from [35,36]). Cronbach alpha for the PA action planning items was .96.

#### Action Control

Action control in terms of PA was measured with four items using 5-point Likert scales, such as “During the last month, I have constantly monitored my...” Response options ranged from 1=definitely not to 5=definitely. Subcategories referred to self-monitoring of PA, awareness of PA standards, self-regulatory effort to be more physically active, and self-regulatory effort to conform to norm behavior (eg, to be moderately active) (derived from [39]). Cronbach alpha for the PA action control items was .94.

### Demographics

Items regarding gender, age, BMI, educational level, cultural background, accommodation, and living situation were included at the beginning of the baseline measure. Ethnicity was defined according to the procedures of Statistics Netherlands; individuals were considered to have a Dutch background if both parents were born in the Netherlands. If one of the parents was born outside the Netherlands, the student was considered to have a non-Dutch background [40].

### Self-Reported Intervention Evaluation

To evaluate subjective experience of using the Balance It app or website, 19 items regarding the Balance It app in general were preceded by the stem: “What did you think of...” Response options ranged from 1 (very bad) to 5 (very good) (compare attitude measures [35,36]). Cronbach alpha for attitude toward the Balance It app in general was .98. The 13 items were preceded by the same stem and referred to the specific game elements included (eg, “What did you think of the theme of Balance It?). Response options ranged from 1 (very stupid) to 5 (very funny) (compare attitude measures [35,36]). Cronbach alpha for the attitude toward game elements was .98.

### Statistical Analyses

Descriptive statistics were used to characterize both study groups at baseline (ie, gender, age, educational level, ethnicity, and and body mass index [BMI]). Chi-square tests and *t* tests were conducted to evaluate whether participant characteristics were related to drop out during the study. Because there was no significant differentiation between school levels, linear regression analyses were performed to study dietary intake and PA change over time, differences between the intervention and control groups, and differences between active users and the control group. In these analyses, primary outcomes were analyzed and differences between groups on determinants of the primary outcomes were explored (controlling for condition and baseline differences). After doing the linear regression analyses, multiple testing adjustment procedures were taken into account according to the Benjamini-Hochberg procedures (ie, we calculated the false discovery rates [FDR] for all primary outcomes and exploratory determinants of these outcome measures). A *P* value of .05 or lower was considered to be statistically significant. All analyses were conducted with IBM SPSS version 20.0 (IBM Corporation).

## Results

### Participants and Dropout Analysis

**Figure 2:**
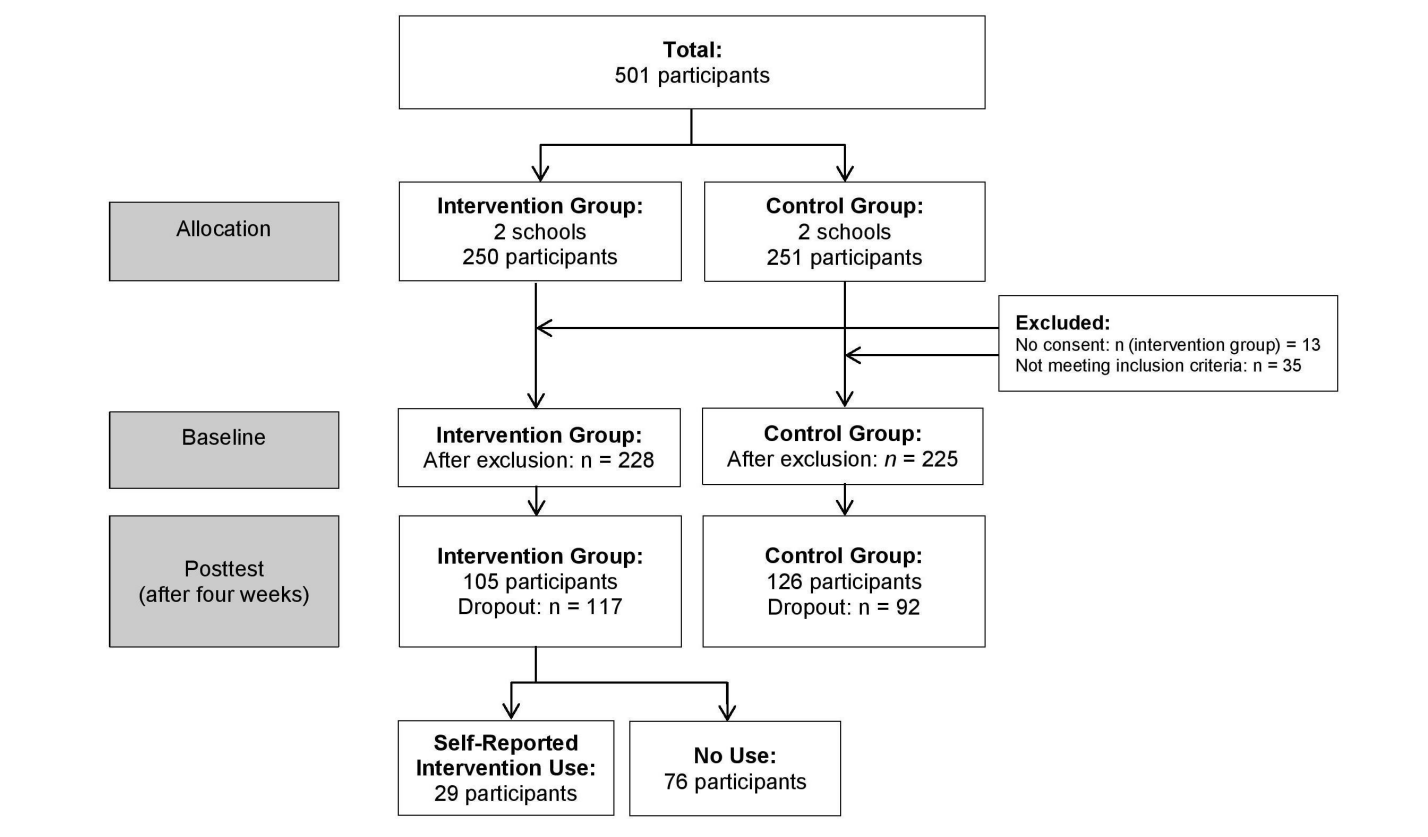
Flow diagram of the enrollment and selection of study participants.

In total, 501 students were invited to participate in this study (intervention: n=250; control: n=251; Figure 2). Of all students invited, 488 participated (97.4%). We excluded 6 students because they were younger than 15 years and 29 students because they were older than 21 years. After exclusion, 228 participants in the intervention group and 225 in the control group remained at baseline. After 4 weeks, 117 participants dropped out from the intervention group, and 92 participants dropped out from the control group. Logistic regression analyses revealed that participants who dropped out were significantly older (mean 17.69, SD 1.53 years) than nondropouts (mean 17.28, SD 1.26 years; OR 0.81, 95% CI 0.71-0.93). Tests also showed that students with a non-Dutch background were more likely to drop out (119/209, 56.9%) than students with a Dutch background (86/209, 41.1%; OR 1.95, 95% CI 1.31-2.92; unknown background: 4/209, 1.9%). We did not find any significant differences in terms of gender, level of education, year of education, or BMI. The BMI distribution of the sample was comparable with previous research on secondary vocational education students’ health and weight [41]. At final count, 105 participants were included in the intervention group and 126 participants were included in the control group.

#### Active Users

Of all participants who remained in the intervention group at posttest (n=105), 27.6% (29/105) reported actual intervention use. Compared to the control group (n=200), self-reported active users were less likely to follow vocational education related to care and well-being (*P*<.001) and more likely to follow vocational education in economics (*P*<.001). Active users were also more likely to be in their first year (n=100) as compared to the control group (ie, n=124; *P*=.01). The two groups did not significantly differ in age, gender, ethnicity, BMI, social vocational education sector, technique vocational education sector, or level of education.

### Baseline Between-Group Differences

Table 1 presents the demographic background of Balance It participants at baseline (N=231). Compared to the control group (mean 17.52, SD 1.36 years), participants in the intervention group were younger (mean 16.96, SD 1.10 years; *P*=.05). They were also more likely to participate in the economics vocational education sector and less likely to participate in care and well-being, social work, and economy vocational education sectors. Finally, they were more likely to be in the first year of secondary vocational education. Therefore, we included these variables as covariates in all further analyses. We also controlled for baseline differences between the intervention group and the control group in case they differed in behavioral outcome and determinant measures. As such, the intervention group at baseline was more likely to use active transport as compared to the control group (*P*=.04). Therefore, we controlled for the use of active transport at baseline in further analyses concerning active transport.

**Table 1:** Demographic background of Balance It participants at baseline (N=231).

Demographic variables		Intervention group (n=105)	Control group (n=126)	χ^2^ (df)	*t*_227_	*P* value
Age (years), mean (SD)		16.96 (1.10)	17.52 (1.36)		–3.34	.003
Gender (male), n (%)		39 (37.1)	47 (37.3)	0.0 (1)		.93
Ethnicity (Dutch), n (%)		77 (73.3)	92 (73.0)	0.1 (1)		.77
**Vocational education sector track, n (%)**						
	Care and well-being	25 (23.8)	115 (91.3)	108.4 (1)		.001
	Economics	71 (67.6)	0 (0.0)	128.9 (1)		.001
	Technique	1 (1.0)	2 (1.6)	0.2 (1)		.69
	Social work	0 (0.0)	1 (1.0)	0.8 (1)		.55
**Educational level, n (%)**				0.5 (1)		.49
	Level 3	5 (4.8)	9 (7.1)			
	Level 4	95 (90.5)	115 (91.3)			
**Year of education, n (%)**				39.3 (1)		.001
	Year 1	100 (100.0)	84 (67.7)			
	Year 2	0 (0.0)	40 (34)			
**Living situation**				5.3 (4)		.26
	Both parents	79 (76.7)	100 (79.4)			
	One parent	19 (18.4)	16 (12.7)			
	Alone	2 (1.9))	2 (1.6)			
	Other	3 (2.9)	8 (6.3)			
**BMI categories, n (%)**				4.7 (3)		.66
	Underweight (BMI <18.5)	8 (12.7)	10 (11.2)			
	Normal weight (BMI 18.5-25)	47 (74.6)	58 (65.1)			
	Overweight (BMI 25-30)	8 (12.7)	18 (20.2)			
	Obese (BMI >30)	0 (0.0)	3 (3.4)			

**Table 2:** Effects of Balance It on behavioral outcomes and determinants.^a^

Outcome variable		T0, mean (SD)		T1, mean (SD)		T0-T1, ∆ mean		Difference test	
		Intervention (n=103)^b^	Control (n=125)^c^	Intervention (n=103)^d^	Control (n=125)^e^	Intervention	Control	B (95% CI)	*R*^2^ change
**Behavioral outcomes**									
	Fruit intake (mean portion/day)	0.81 (0.68)	0.80 (0.68)	1.05 (0.75)	0.81 (0.62)	0.14	0.01	0.21 (–0.07 to 0.49)	.01
	Vegetable intake (mean portion/day)	1.26 (0.33)	1.32 (0.38)	1.21 (0.41)	1.28 (0.36)	–0.05	–0.04	–0.03 (–0.15, 0.10)	.00
	Snack consumption (mean portion/day)	0.91 (0.50)	0.98 (0.51)	0.86 (0.51)	0.90 (0.48)	–0.05	–0.08	0.01 (–0.17 to 0.19)	.00
	Soft drink consumption (mean portion/day)	1.07 (0.53)	1.11 (0.59)	0.92 (0.57)	1.07 (0.57)	–0.15	–0.04	–0.25 (–0.45 to –0.05)	.03
	Moderate PA (days) ^f^	4.30 (2.41)	3.82 (2.67)	3.91 (2.54)	3.31 (2.51)	–0.39	–0.51	0.20 (–0.87 to 1.27)	.00
	Vigorous PA (days)	5.21 (2.26)	5.25 (2.07)	4.74 (2.47)	4.78 (2.27)	–0.47	–0.47	0.10 (–0.12 to 1.33)	.00
	Active transport (days)	2.55 (1.99)	2.50 (2.37)	3.20 (2.51)	2.38 (2.13)	0.65	–0.12	0.94 (0.06 to 1.81)	.02
**Determinants: fruit and vegetable intake (5-point scale)**									
	Attitude	4.01 (0.53)	3.98 (0.60)	3.93 (0.80)	4.00 (0.64)	–0.08	0.02	–0.26 (–0.51 to –0.02)	.02
	Self-efficacy	4.33 (0.80)	4.29 (0.81)	4.04 (1.02)	4.13 (0.92)	–0.29	–0.16	–0.44 (–0.85 to 0.03)	.02
	Intention	3.86 (1.00)	3.73 (1.05)	3.66 (1.09)	3.67 (1.11)	–0.20	–0.06	–0.32 (–0.74 to 0.09)	.01
	Perceived barriers	3.45 (0.89)	3.52 (0.96)	3.53 (1.03)	3.49 (0.91)	0.08	–0.03	0.13 (–0.24 to 0.51)	.00
	Action planning	3.04 (1.13)	3.05 (1.14)	3.37 (1.05)	2.86 (1.20)	0.33	–0.19	0.36 (–0.10 to 0.82)	.01
	Action control	2.89 (1.26)	2.80 (1.17)	3.40 (1.14)	2.86 (1.20)	0.51	0.06	0.53 (0.04 to 1.02)	.02
**Determinants: snack and soft drink consumption (5-point scale)**									
	Attitude	3.63 (0.66)	3.42 (0.61)	3.72 (0.87)	3.63 (0.67)	0.09	0.19	–0.23 (–0.53 to 0.06)	.01
	Self-efficacy	4.28 (0.77)	4.10 (0.83)	3.92 (1.04)	3.91 (0.94)	–0.36	–0.19	–0.37 (–0.84 to 0.09)	.01
	Intention	3.75 (1.03)	3.40 (1.03)	3.60 (1.05)	3.35 (1.08)	–0.15	–0.05	–0.14 (–0.55 to 0.28)	.00
	Perceived barriers	3.52 (0.98)	3.35 (0.92)	3.48 (1.01)	3.37 (0.89)	–0.04	0.02	0.21 (–0.18 to 0.59)	.01
	Action planning	3.11 (1.13)	2.99 (1.11)	3.37 (1.06)	3.06 (1.07)	0.27	0.07	0.33 (–0.12 to 0.77)	.01
	Action control	2.96 (1.27)	2.78 (1.16)	3.28 (1.15)	2.73 (1.20)	0.32	–0.05	0.48 (–0.01 to 0.97)	.02

**Determinants: PA (5-point scale)**									
	Attitude	4.21 (0.53)	4.21 (0.58)	4.02 (0.85)	4.13 (0.60)	–0.20	–0.09	–0.18 (–0.43 to 0.07)	.01
	Self-efficacy	4.42 (0.66)	4.30 (0.82)	3.98 (1.04)	4.08 (0.89)	–0.44	–0.22	–0.39 (–0.80 to 0.02)	.02
	Intention	4.01 (0.96)	3.87 (1.03)	3.70 (1.07)	3.68 (1.04)	–0.31	–0.19	–0.44 (–0.87 to –0.01)	.02
	Perceived barriers	3.47 (1.02)	3.16 (1.05)	3.39 (1.09)	3.16 (1.04)	–0.08	0.00	–0.03 (–0.40 to 0.35)	.00
	Action planning	3.26 (1.17)	3.21 (1.08)	3.43 (1.06)	3.16 (1.06)	0.17	–0.05	0.27 (–0.19 to 0.74)	.01
	Action control	3.05 (1.26)	2.93 (1.16)	3.38 (1.09)	2.82 (1.16)	0.33	–0.11	0.60 (0.15 to 1.04)	.03

^a^ Differences between the intervention group and control group at posttest measurement are derived via linear regression analyses for linear variables (B and 95% CI are reported), correcting for the baseline score of Y, and demographic variables for which differences were found between groups at baseline (age, vocational education sector, year of education, and the use of active transport); corrected for multiple testing (based on false discovery rate).

^b^ Except for action planning and action control for fruit and vegetable intake, snack and soft drink consumption, and PA (n=99).

^c^ Except for action planning and action control for fruit and vegetable intake, snack and soft drink consumption, and PA (n=124).

^d^ Except for fruit and vegetable intake and snack and soft drink consumption (n=126), moderate PA (n=124), and active transport (n=123) for behavioral outcomes, and as follows for fruit and vegetable intake, snack and soft drink consumption, and PA: attitude (n=99), self-efficacy and intention (n=96), perceived barriers (n=95), and action planning and action control (n=92).

^e^ Except for fruit and vegetable intake and soft drink consumption (n=104), moderate PA (n=101), vigorous PA (n=98), and active transport (n=99) for behavioral outcomes, and action planning and action control for fruit and vegetable intake, snack and soft drink consumption, and PA (n=124).

^f^ Physical Activity.

### Group Comparison: Intervention Versus Control Group

Change scores for the intervention group were compared with change scores for the control group for both behavioral (primary) outcome measures and determinants (secondary outcome measures). All findings of the linear regressions are presented in Table 2.

#### Exploratory Analysis: Primary Outcomes and Determinants of Primary Outcomes

After correcting for multiple testing, we did not find significant differences in change scores between the intervention group and the control group for dietary intake and PA (see Table 2). There were no significant differences in change scores between the two groups on determinants of dietary intake and PA.

#### Exploratory Analyses: Active Users Versus the Control Group

The same regression analyses performed to compare the intervention and control groups were also used to compare the groups “active users” in the intervention group (29/103, 28.2%) and the control group (n=124). Allocation to these groups was based on self-reported intervention use. There were no significant baseline differences between active and nonactive users in the intervention group.

##### Baseline Differences Between Active Users and the Control Group

Compared to the control group, active users were more likely to participate in the economics vocational education sector (χ^2^_1_=90.4, *P*<.001) and active users were more likely to be in the first year of secondary vocational education (χ^2^_1_=12.3, *P*<.001). Therefore, we controlled for these differences in the following analyses. We also controlled for baseline differences between the active users and the control group in case they differed significantly on primary behavioral outcomes and exploratory determinant measures. We found that the active users at baseline were more likely to use active transport as compared to the control group (*P*=.04). Therefore, we controlled for the use of active transport at baseline in all subsequent analyses concerning active transport.

##### Exploratory Analysis: Active Users Versus the Control Group

After correcting for multiple testing, we found that active users reported marginally stronger increases in fruit intake (active users: mean change=0.51; control group: mean change=0.01; beta=0.34, *P*=.06, *R*^2^ change=.02), stronger decreases in snack consumption (active users: mean change=–0.20; control group: mean change=–0.08; beta=–0.36, *P*=.01, *R*^2^ change=.05), and stronger increased use of active transport (active users: mean change=0.92; control group: mean change=–0.12; beta=1.58, *P*=.02, *R*^2^ change=.03). In terms of unhealthy eating, results also revealed significant differences in action planning (active users: mean change=0.42; control group: mean change=0.07; beta=0.91, *P*=.01, *R*^2^ change=.04), and PA (active users: mean change=0.44; control group: mean change=–0.05; beta=0.83, *P*=.03, *R*^2^ change=.03), and action control (active users: mean change=0.63; control group: mean change=–0.05; beta=1.25, *P*=.001, *R*^2^ change=.08).

### Process Evaluation of Self-Reported and Registered Game Play

User data (an objective measure) showed that Balance It was played 771 times in total. These games primarily consisted of daily tasks (671/771, 87.0%) and individual game play (632/771, 82.0%). Of all the goals set (ie, type of tasks), players chose to improve their fruit intake in 15.0% of all cases (116/771 of which 44.0%, 51/116 of the goals were accomplished), 3.0% opted to increase their vegetable intake (23/771 of which 39%, 9/23 of the goals were accomplished), 29.1% opted to decrease their snack consumption (224/771 of which 70.1%, 157/224 of the goals were accomplished), 8.9% opted to decrease their soft drink consumption (69/771 of which 63%, 44/69 of soft drink-related goals were accomplished), 31.0% opted to increase their moderate PA (239/771 of which 54.8%, 131/239 of moderate PA goals were accomplished), and 13.0% opted to increase their vigorous PA (100/771 of which 39%, 39/100 of vigorous PA goals were accomplished). Goal accomplishment was more likely when participants were motivated (OR 2.6, 95% CI 1.9-3.5), and less likely when they did not have the time (OR 0.6, 95% CI 0.4-0.9) or when they experienced the location as a barrier (OR 0.6, 95% CI 0.4-0.9).

At posttest, 50% (15/29) of the participants who used the intervention reported that they played Balance It because they wished to have a healthier lifestyle, and 50% (14/29) played the game because they were asked to for the purpose of our study (29/103). Of the participants who did not play Balance It, 24% (18/74) reported that they did not have the time to play. The participants who used the intervention were, on average, neutral to positive about the Balance It app. When asked whether they were planning to recommend Balance It to others, participants gave a mean score of 3.14 (SD 1.03) on a scale ranging from 1 (very bad) to 5 (very good); likewise, the mean rating for the tutorial (using the same scale) was 3.72 (SD 0.75). Also, the specific game elements were evaluated neutrally to positively, on average, ranging from 3.43 (SD 1.00) on a scale ranging from 1 (very stupid) to 5 (very nice) for the construction worker, to 3.62 (SD 0.90) for the option of using special powers on the tower of an opponent. The mean overall rating given for the Balance It app (on a scale of 1 to 10, 1=the lowest grade, 10=the highest grade) was 6.71 (SD 1.96). The mean overall rating for the website (using the same scale) was 6.50 (SD 1.40).

## Discussion

The aim of this study was to pilot the effects of Balance It, a serious game intervention targeting secondary vocational education students’ dietary intake and PA.

### Main Findings

No significant differences between the intervention and control groups in terms of dietary intake and PA (the primary outcomes) were observed. Additional exploratory analyses did not reveal significant differences in change scores between the intervention and control group in terms of psychological determinants of dietary intake and PA, as targeted by Balance It.

The study also revealed that the number of people that used the Balance It intervention was less than expected because only 27.6% used it as intended. For exploratory purposes, we examined the potential of Balance It among active users by comparing participants in the intervention group who reported that they had used the intervention with the control group. We did find that active users increased their fruit consumption marginally and active transport significantly, and showed stronger decreases in snack consumption compared to the control group. Although we should acknowledge that other factors could explain these differences (ie, self-selection), the findings could indicate Balance It may contribute to changes in PA and dietary intake if used as planned.

Taking into account that a difference of 100 kcal in daily caloric intake/expenditure can contribute to overweight prevention [42], the increase in active transportation by 0.92 days on average may contribute to the prevention of overweight. Snack consumption was only decreased by a mean 0.20 snack portions per day, which may not be sufficient to make a change in daily energy balance. Nevertheless, active users showed an improvement in action planning and action coping skills to decrease their snack consumption, which may be related to students’ increase in fruit consumption as a healthy alternative to unhealthy snacks. Nevertheless, the changes observed may not be large enough to prevent overweight. In general, active users rated the intervention moderately positively and registered data showed that active users mainly opted to decrease their snack consumption (29.1%) or to increase their moderate PA (31.0%).

Consistent with previous serious gaming studies targeting dietary intake and PA, our results suggest that the use of a self-regulation game intervention could improve dietary intake and active transport among youth [24,43]. Despite the significant increase in use of active transport for the intervention users, they did not report a significant increase in moderate or vigorous PA. That we found no direct effect of the intervention on PA may be because posttest measures took place after 4 weeks of game play, whereas PA goals were partially set on a weekly basis, taking up to 6 weeks of game play, and therefore were not yet completed at the time of the posttest measure. The 4-week period may also be too short to expect change in PA [44].

Previous research shows that youth from low SES families are less engaged with health behaviors and not as successful in terms of translating their health intentions into behavior [45]. Therefore, we incorporated gameplay, reminders, professional support, and social contracting into our serious game to stimulate intervention use. Moreover, we plan to embed the intervention within an existing student tracking system as suggested by Crutzen and other authors [46-48] (see [30] for a more detailed description of Intervention Mapping step 5: program implementation) to stimulate initial use. Despite these attempts to encourage implementation use, only 27.6% of the intervention group reported actual use of Balance It. Although these active users were moderately positive about Balance It, it may be that players were not sufficiently transported into the game by the narrative of Balance It because the narrative that was used to stimulate immersion is more in line with what Lu et al [49] describe as an “instruction,” which does not adequately facilitate immersion. According to Lu et al, narratives should have attractive features (eg, a plot, a beginning, middle, and an end), and should allow players to experience a character’s happiness on their journey toward adoption of a healthy behavior more directly and vividly than didactic instruction alone [49]. However, in practice, professional game designers often stress the importance of game simplicity to enhance motivation to play (see also [50]), limiting the space for extensive narratives to be included within a game. One way of resolving this issue would be to use so-called novellas, which can be defined as highly immersive stories designed to increase engagement with the game intervention provided prior to the game [51]. Placing novellas outside the Balance It app may also be beneficial in terms of exposure because students were stimulated to follow the link to the Balance It website by the research assistant during pretest, but the research assistant was not able to check if all students downloaded the app. As such, participants would be more likely to be exposed to the novella, increasing the likelihood of intervention (or Balance It app) use. These so-called “novellas” are still in their infancy, but because they seem to be a rather promising way of countering low levels of engagement, further research is recommended.

### Limitations

Some limitations of this study should be acknowledged. First, the study is a cluster randomized trial, which was chosen over a randomized controlled trial because of practical considerations (ie, school coordinators who wanted their students to be in the same condition), to prevent contamination effects and to enhance participant compliance [31,52]. It should also be noted that participants who were at criteria for the outcomes (ie, students who already eat healthy and have sufficient PA) at baseline were included in the cluster randomized trial, which may have reduced the overall effect size. A second limitation is that only a pretest and posttest were included in this study, and follow-up measures to evaluate long-term effects were lacking. Consequently, the results of this study are based on change scores collected over a 4-week period, whereas many PA-related goals that were set by game players took 6 weeks to accomplish. As such, we might not expect the full effects of Balance It to reveal themselves until participants had used it for 6 weeks. Participants were contacted via email and requested to fill in the follow-up questionnaire 4 weeks after the posttest measure, but with a response rate of only 4%, we did not analyze these data. Finally, email addresses were inaccurately reported (or not reported) in the Balance It app, which was unfortunate because we needed them to merge (objective) user data with self-reported survey data at baseline and at posttest. Consequently, user data could not be merged with self-reported data and conclusions about the objective use of the intervention in relation to the learning outcomes should be interpreted with care. We recommend that future research use a randomized controlled trial design and incorporate appropriate follow-up measures and checks regarding the collection of email addresses or the inclusion of other merge variables.

Finally, it should be noted that despite the potential of the peer-support component as included in the Balance It website to increase intervention effects on self-regulation skills [53], usage of the peer-support system was rather disappointing (ie, only 7% of the users reported actual use of the peer-support system). The lack of peer-support system use is a commonly reported problem in online interventions [54], although if used, peer-support systems could have facilitated behavior change and problem-solving processes [29]. Because the peer-support system was placed outside the Balance It app as a result from a trade-off that was made between developers and behavior change experts about the number of layers within the game, visiting the system online may have been a barrier. A second explanation for the lack of peer-support system use may also be derived from the limited number of schools (ie, n=4) included in this cluster randomized trial. In total, only two schools were allocated to the intervention group, indicating that most participants knew one another in daily life. If students preferred to receive or provide social support, this may have taken place in real life instead of through the online peer-support system of Balance It.

### Conclusion

The Balance It intervention did not show favorable effects on dietary intake and PA compared to the control condition. However, only a small number of people in the intervention condition actually used Balance It (27.6%). Exploratory analyses did suggest that, if used as planned, Balance It could contribute to changing dietary intake and PA behaviors, albeit it remains debatable whether this would be sufficient to prevent overweight.

## Abbreviations

BMI: body mass index
OR: odds ratio
PA: physical activity
SES: socioeconomic status
